# Supplementary material for: Causal Relationship between Aging and Anorexia Nervosa: A White-Matter-Microstructure-Mediated Mendelian Randomization Analysis
Source: Biomedicines. 2024 Aug 16;12(8):1874. doi: 10.3390/biomedicines12081874 (PMC11351342; doi:10.3390/biomedicines12081874)
Supplement: Supplementary file 1 [file biomedicines-12-01874-s001.zip › Supplementary Figure/Figure S4/Singe SNP plot of MR analysis for TL to ubm-b-1671.pdf]

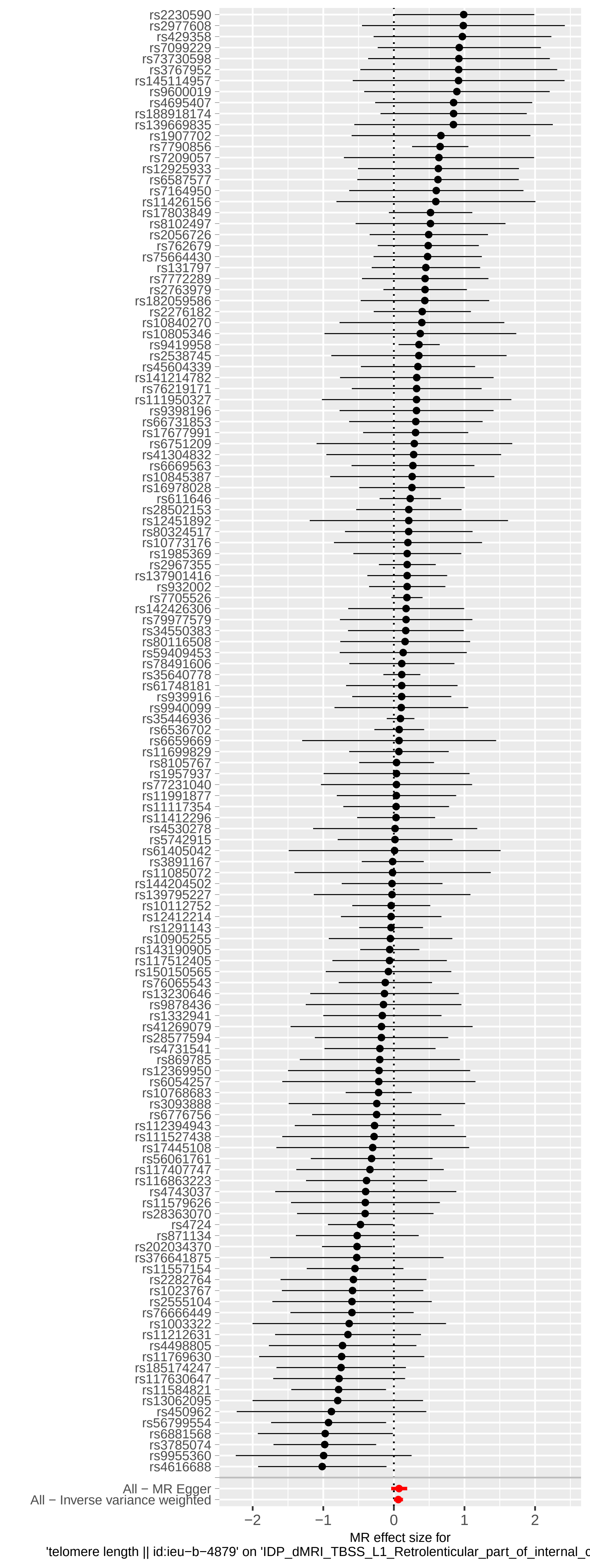
rs2230590
rs2977608
rs429358
rs7099229
rs73730598
rs3767952
rs145114957
rs9600019
rs4695407
rs188918174
rs139669835
rs1907702
rs7790856
rs7209057
rs12925933
rs6587577
rs7164950
rs11426156
rs17803849
rs8102497
rs2056726
rs762679
rs75664430
rs131797
rs7772289
rs2763979
rs182059586
rs2276182
rs10840270
rs10805346
rs9419958
rs2538745
rs45604339
rs141214782
rs76219171
rs111950327
rs9398196
rs66731853
rs17677991
rs6751209
rs41304832
rs6669563
rs10845387
rs16978028
rs611646
rs28502153
rs12451892
rs80324517
rs10773176
rs1985369
rs2967355
rs137901416
rs932002
rs7705526
rs142426306
rs79977579
rs34550383
rs80116508
rs59409453
rs78491606
rs35640778
rs61748181
rs939916
rs9940099
rs35446936
rs6536702
rs6659669
rs11699829
rs8105767
rs1957937
rs77231040
rs11991877
rs11117354
rs11412296
rs4530278
rs5742915
rs61405042
rs3891167
rs11085072
rs144204502
rs139795227
rs10112752
rs12412214
rs1291143
rs10905255
rs143190905
rs117512405
rs150150565
rs76065543
rs13230646
rs9878436
rs1332941
rs41269079
rs28577594
rs4731541
rs869785
rs12369950
rs6054257
rs10768683
rs3093888
rs6776756
rs112394943
rs111527438
rs17445108
rs56061761
rs117407747
rs116863223
rs4743037
rs11579626
rs28363070
rs4724
rs871134
rs202034370
rs376641875
rs11557154
rs2282764
rs1023767
rs2555104
rs76666449
rs1003322
rs11212631
rs4498805
rs11769630
rs185174247
rs117630647
rs11584821
rs13062095
rs450962
rs56799554
rs6881568
rs3785074
rs9955360
rs4616688
All − MR Egger
All − Inverse variance weighted
−2
−1
0
1
2
MR effect size for
'telomere length || id:ieu−b−4879' on 'IDP_dMRI_TBSS_L1_Retrolenticular_part_of_internal_c
